# Development and Validation of a Machine Learning–Based Prediction Model for Cardiovascular Disease in Patients with Metabolic Dysfunction–Associated Fatty Liver Disease

**DOI:** 10.5152/tjg.2026.25611

**Published:** 2026-02-13

**Authors:** Li-Huan Wang

**Affiliations:** Department of Infectious Diseases, Jiashan County First People’s Hospital, Jiashan, China

**Keywords:** Cardiovascular disease, machine learning, MAFLD, NHANES, risk stratification, SHAP

## Abstract

**Background/Aim:**

Metabolic dysfunction–associated fatty liver disease (MAFLD) is strongly associated with increased cardiovascular disease (CVD) risk. However, traditional cardiovascular risk assessment tools may inadequately capture the complex pathophysiology linking hepatic and CVD in MAFLD patients. This study aimed to develop and validate machine learning models to predict CVD risk in MAFLD patients.

**Materials and Methods:**

: This cross-sectional study analyzed NHANES 2017-2023 data, with participants randomly split into training (70%) and validation (30%) sets.

**Results:**

: A total of 6828 adults with MAFLD were included (13.1% with prevalent CVD). Least Absolute Shrinkage and Selection Operator regression identified 14 key predictors, with age, nonalcoholic fatty liver disease Fibrosis Score (NFS), and albumin emerging as the most influential. Critically, liver-specific markers (NFS: mean |SHAP| = 0.0299; albumin: 0.0289) demonstrated superior predictive capacity compared to traditional cardiovascular risk factors (hypertension: 0.0145; diabetes: 0.0107; smoking: 0.0035). SHapley Additive exPlanations analysis revealed that older age, higher NFS (particularly > −1.0), and lower albumin (<3.5 g/dL) were the strongest drivers of CVD risk, with NFS showing clear threshold effects.

**Conclusion:**

: The findings confirm that traditional cardiovascular risk assessment approaches are insufficient for MAFLD patients, as liver-specific markers—particularly hepatic fibrosis (NFS) and liver synthetic function (albumin)—dominated cardiovascular risk prediction over conventional risk factors (hypertension, diabetes, smoking). This paradigm shift underscores the necessity of integrated liver-heart assessment in MAFLD and supports routine hepatic fibrosis evaluation for cardiovascular risk stratification. The model demonstrates immediate clinical applicability through existing electronic health records, enabling early identification of high-risk patients for targeted preventive interventions.

**Main Points**

- Fifteen different machine learning algorithms were systematically evaluated using LASSO regression to develop a cardiovascular disease (CVD) prediction model for metabolic dysfunction–associated fatty liver disease.
- The SHapley Additive exPlanations analysis demonstrated that the nonalcoholic fatty liver disease (NAFLD) Fibrosis Score and albumin levels dominated risk prediction to CVD in NAFLD.
- The model demonstrates excellent calibration across all probability ranges and substantial clinical utility via decision curve analysis.

## Introduction

Metabolic dysfunction–associated fatty liver disease (MAFLD) has emerged as the leading cause of chronic liver disease globally, affecting approximately a quarter of the world’s adult population.^1^^,^^2^ Unlike nonalcoholic fatty liver disease (NAFLD), MAFLD positively defines the disease based on hepatic steatosis coupled with metabolic dysregulation, without requiring the exclusion of other chronic liver diseases or alcohol intake.^1^^,^^3^ This definition highlights MAFLD as a multi-systemic disorder closely linked with metabolic comorbidities such as type 2 diabetes mellitus, hyperlipidemia, and hypertension.^2^^,^^4^^,^^5^ Critically, individuals with metabolically unhealthy MAFLD face significantly higher risk of major adverse cardiovascular events, all-cause mortality, and cardiovascular disease (CVD) mortality compared to non-MAFLD individuals.^6^ Recent epidemiological studies have confirmed the high prevalence of MAFLD among type 2 diabetes patients, with rates approaching 70% in some populations, alongside substantial rates of advanced fibrosis risk.^7^ Given that CVD remains a primary global health threat, the strong association between MAFLD and increased cardiovascular risk underscores an urgent need for effective strategies to identify and manage high-risk patients.^8^^,^^9^

Despite growing recognition of MAFLD’s systemic impact, substantial heterogeneity persists within the patient population, driven by varying metabolic burdens.^6^ This presents significant challenges in accurately stratifying cardiovascular risk, making early interventions difficult to implement. Traditional clinical assessment often relies on subjective judgment and lacks objective, quantifiable tools for precise risk prediction.^10^ However, rapid advancements in machine learning (ML) offer promising solutions.^11^ The ML models excel at extracting complex patterns from large datasets and have been successfully applied to predict depression risk in patients with cardiovascular metabolic diseases and to develop screening nomograms for MAFLD.^10^ By leveraging these capabilities, the identification of MAFLD patients at elevated risk for developing CVD could be significantly improved.

Using National Health and Nutrition Examination Survey (NHANES) data,^12^ 15 ML algorithms were systematically evaluated with LASSO-guided feature selection. The analysis focused on easily accessible clinical variables and incorporated SHapley Additive exPlanations (SHAP) to enhance model interpretability. Through rigorous validation, robust predictive models were constructed, and key clinical predictors facilitating early risk stratification in this vulnerable population were identified.

## Materials and Methods

### Study Design and Participants

This cross-sectional study utilized data from the NHANES 2017-2023. The NHANES is a nationally representative survey employing complex, multistage probability sampling to assess health and nutritional status of the civilian, non-institutionalized United States population. The survey combines comprehensive interviews with standardized physical examinations and laboratory tests in mobile examination centers. As this study involved publicly available, de-identified data, additional institutional review board approval was not required. Informed consent was not required as well, due to publicly available, de-identified data in this study.

The initial dataset comprised 36 747 participants. Supplementary Figure 1 illustrates the selection process. Participants younger than 18 years (n = 13 045), those with missing liver ultrasound data (n = 4204), pregnant women (n = 186), individuals with unknown CVD status (n = 230), participants with missing key laboratory covariates including high-density lipoprotein (HDL) cholesterol, total cholesterol, uric acid, or creatinine (n = 3378), and those not meeting MAFLD diagnostic criteria (n = 4598) were excluded. The final cohort consisted of 6828 adults with confirmed MAFLD, including 896 (13.1%) with prevalent CVD and 5932 (86.9%) without CVD.

### Definitions of Metabolic Dysfunction−Associated Fatty Liver Disease

Following international consensus guidelines, MAFLD was diagnosed based on hepatic steatosis with metabolic abnormalities.^1^ Hepatic steatosis was diagnosed exclusively using vibration-controlled transient elastography with Controlled Attenuation Parameter ≥ 248 dB/m, which represents the only objective hepatic steatosis assessment available in NHANES 2017-2023. This threshold has been validated in large meta-analyses for detecting hepatic steatosis with sensitivity of 69% and specificity of 82% for any grade of steatosis.^13^ MAFLD diagnosis required at least one of the following criteria: overweight/obesity (body mass index (BMI) ≥25 kg/m^2^), type 2 diabetes, or evidence of metabolic dysregulation. Metabolic dysregulation was defined by the presence of at least two of the following: waist circumference ≥102 cm in men or ≥88 cm in women, blood pressure ≥130/85 mmHg or antihypertensive medication use, triglycerides ≥1.70 mmol/L or lipid-lowering therapy, HDL cholesterol <1.0 mmol/L in men or <1.3 mmol/L in women, prediabetes, Homeostatic Model Assessment of Insulin Resistance (HOMA-IR) ≥2.5, or high-sensitivity C-reactive protein >2 mg/L.

### Definitions of Cardiovascular Disease

Cardiovascular disease was defined as a composite outcome based on self-reported physician diagnosis of any of the following conditions: coronary heart disease, angina pectoris, myocardial infarction, stroke, or congestive heart failure. This definition was ascertained through standardized questionnaire responses during the NHANES interview process.

### Assessment of Covariates

Covariates were selected based on established associations with cardiovascular outcomes in MAFLD patients. Demographic variables included age, sex, race/ethnicity (Mexican American, Non-Hispanic Black, Non-Hispanic White, and Other), education level, and family poverty-income ratio (PIR). The PIR, calculated by NHANES as the ratio of family income to the federal poverty threshold (adjusted for family size, composition, and survey year), was utilized as a continuous variable ranging from 0 to 5. Anthropometric measurements comprised BMI, waist circumference, and A Body Shape Index (ABSI) calculated as waist circumference/(BMI^2/3^ × height^1/2^).^14^ Blood pressure measurements included systolic (SBP) and diastolic blood pressure (DBP), averaged from 3 consecutive readings after a 5-minute rest. Laboratory parameters encompassed glycated hemoglobin (HbA1c), liver enzymes (alanine aminotransferase (ALT), aspartate aminotransferase (AST), alkaline phosphatase, Gamma-glutamyl transferase), albumin, total bilirubin, serum creatinine, blood urea nitrogen, uric acid, total cholesterol, HDL cholesterol, high-sensitivity C-reactive protein, and complete blood count parameters (white blood cells, lymphocytes, monocytes, neutrophils, platelets). Clinical indices included estimated glomerular filtration rate (eGFR), estimated glucose disposal rate (eGDR), Fibrosis-4 index (FIB-4), and NAFLD Fibrosis Score (NFS). Lifestyle factors included smoking status, alcohol consumption, diabetes, prediabetes, hypertension, chronic obstructive pulmonary disease (COPD), chronic kidney disease (CKD), and malignancy history.

Validated clinical indices were calculated as follows. Estimated glomerular filtration rate was determined using the CKD-EPI equation.^15^ Estimated glucose disposal rate was calculated as 21.16 − (0.09 × waist circumference) − (3.41 × hypertension status) − (0.55 × HbA1c).^16^ The FIB-4 index was computed as (age × AST) / (platelet count × √ALT). The NFS was calculated as −1.675 + (0.037 × age) + (0.094 × BMI) + (1.13 × diabetes status) + (0.99 × AST/ALT ratio) − (0.013 × platelet count) − (0.66 × albumin).^17^ Comorbidities were defined as: diabetes (HbA1c ≥ 6.5%, fasting glucose ≥ 126 mg/dL, or medication use), prediabetes (HbA1c 5.7%-6.4% or fasting glucose 100-125 mg/dL), hypertension (SBP ≥ 140 mmHg, DBP ≥ 90 mmHg, or medication use), CKD (eGFR<60 mL/min/1.73m^2^ or albumin-to-creatinine ratio ≥30 mg/g), with COPD and malignancy based on self-reported diagnosis. Smoking status and alcohol consumption were categorized by standardized criteria.

### Pre-processing of Machine Learning Features

Missing data can introduce bias and reduce statistical power. Supplementary Figure 2 shows the distribution of missing values across variables. Most variables had <1% missing data, though several showed substantial missingness: marital status (61.4%), HOMA-IR (51.1%), and fasting laboratory parameters (51.1%-51.8%). For variables with missing values deemed clinically essential, random forest imputation was performed using the missForest function in R.^18^ This non-parametric approach captures complex interactions and non-linear relationships, outperforming traditional imputation methods for NHANES data structures.

The complete dataset of 6828 participants was randomly divided into training (70%, n = 4780) and validation (30%, n = 2048) sets using stratified sampling to maintain balanced CVD prevalence (13.1%) across both subsets. To identify relevant predictors while avoiding overfitting, Least Absolute Shrinkage and Selection Operator (LASSO) regression was implemented using the “glmnet” package in R. The LASSO performs variable selection and regularization by shrinking coefficients, effectively removing irrelevant features. The optimal regularization parameter (lambda) was determined through 10-fold cross-validation on the training set. Lambda.1se was selected to achieve better generalization with fewer variables while maintaining comparable predictive performance.

### Development and Validation of 15 Machine Learning Prediction Models

Following LASSO feature selection, 15 ML algorithms were developed to predict CVD risk in MAFLD patients using the caret package in R, with specialized packages for CatBoost and LightGBM. All models underwent hyperparameter optimization through grid search with 5-fold cross-validation on the training set.

The implemented algorithms included tree-based methods (Random Forest, Gradient Boosting Machine, XGBoost, LightGBM, CatBoost, and AdaBoost), linear approaches (Logistic Regression, LASSO, Linear Discriminant Analysis), instance-based learning (K-Nearest Neighbors), kernel methods (Support Vector Machine (SVM) with radial basis function), neural networks (Multi-Layer Perceptron), probabilistic classifiers (Naive Bayes), and dimension reduction techniques (Partial Least Squares). Random Forest aggregates multiple decision trees through bootstrap aggregation. Gradient Boosting variants sequentially build trees to minimize prediction errors with implementation-specific optimizations. Support Vector Machine maps data into higher-dimensional space for non-linear classification. The LASSO incorporates L1 regularization for feature selection. Neural networks capture non-linear relationships through interconnected nodes with activation functions.

Model performance was evaluated using area under the receiver operating characteristic curve (AUC-ROC), accuracy, sensitivity, specificity, positive predictive value, negative predictive value, F1 score, and Youden’s index. Calibration curves assessed agreement between predicted probabilities and observed outcomes. Decision curve analysis evaluated clinical utility by calculating net benefit across probability thresholds. The model with highest validation AUC while maintaining good calibration and clinical utility was selected. SHapley Additive exPlanations analysis enhanced interpretability by quantifying feature contributions to predictions.

### Statistical Analysis

Continuous variables were assessed for normality using the Shapiro–Wilk test. Normally distributed variables are presented as mean ± standard deviation and compared using Student’s *t*-test, while non-normally distributed variables are expressed as median (interquartile range) and analyzed using the Mann–Whitney *U*-test. Categorical variables are presented as frequencies and percentages and compared using the chi-squared test or Fisher’s exact test.

Model performance was evaluated using area under the receiver operating characteristic curve (AUC-ROC) with 95% CIs using the pROC package, along with accuracy, sensitivity, specificity, positive predictive value, negative predictive value, F1 score, and Youden’s index. Calibration was assessed through calibration plots using the rms package and quantified by Brier score. Decision curve analysis was implemented using the rmda package to evaluate clinical utility by calculating net benefit across probability thresholds. Model interpretability was enhanced using SHAP through the shapviz package. All statistical analyses were performed using R software version 4.3.0 (The University of Auckland; Auckland, New Zealand), with 2-sided tests and significance set at *P* < .05.

## Results

### Characteristics of the Participants

A total of 6828 adults with MAFLD were included, randomly divided into training (n = 4780, 70%) and validation (n = 2,48, 30%) sets. Table 1 presents baseline characteristics. Mean age was 53.19 ± 16.19 years (training) and 53.58 ± 15.76 years (validation) (*P* = .361), with males comprising 51.99% and 54.25%, respectively (*P* = .086). Mean BMI exceeded 32 kg/m^2^ in both cohorts. Non-Hispanic White individuals represented the largest racial group (35.31% training, 34.57% validation). Diabetes was present in approximately 30% of participants, with an additional 42% having prediabetes. Hypertension affected over half (54.83% training, 55.27% validation). Median NFS was −1.07 (−2.18, 0.01) and −0.98 (−2.07, 0.06), respectively (*P* = .047). Most baseline characteristics showed no significant differences between sets, confirming appropriate randomization for model development.

### Selection of Main Predictors of Cardiovascular Disease

**Figure 1.**
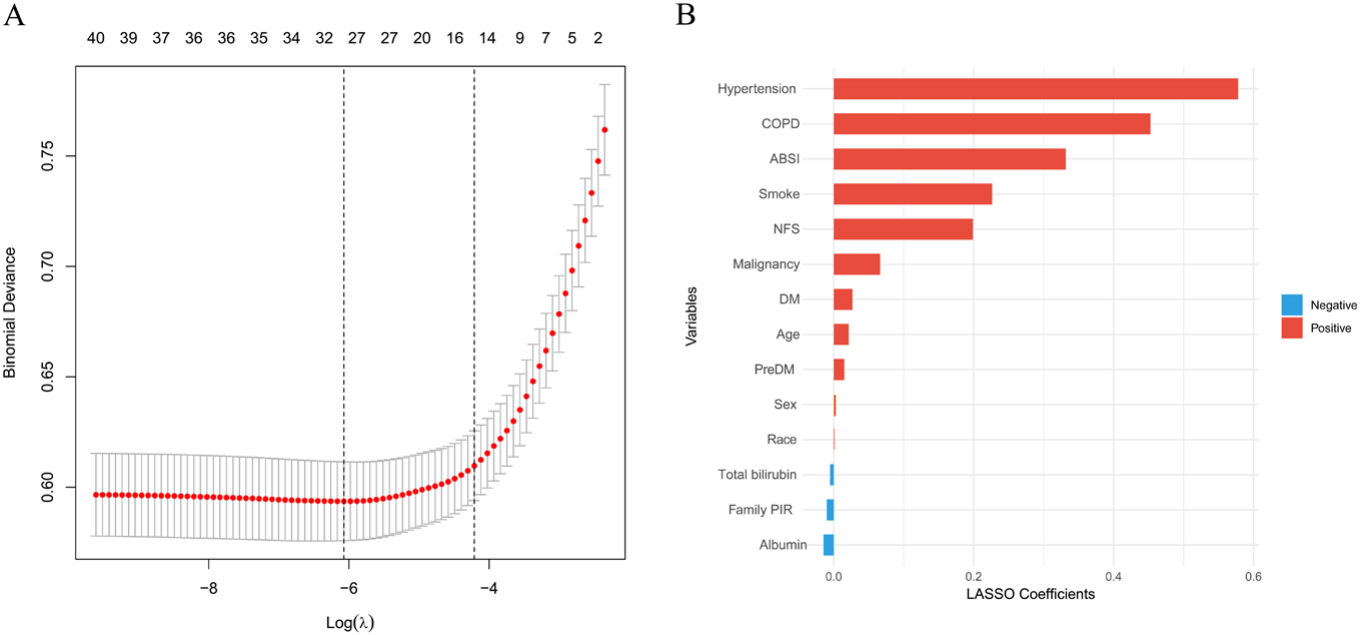
Feature selection using LASSO regression for CVD prediction in MAFLD patients. (A) Cross-validation curve showing binomial deviance as a function of log(lambda). The dotted vertical lines indicate lambda.min (left) and lambda.1se (right), with lambda.1se selected for the most parsimonious model within 1 standard error of minimum cross-validated error. Numbers above the plot indicate the number of non-zero coefficients at each lambda value. (B) LASSO coefficient plot displaying the 14 retained predictors with non-zero coefficients, colored by direction of association (red for positive, blue for negative). The selected variables included both traditional cardiovascular risk factors and MAFLD-specific markers.

Feature selection was conducted using LASSO regression via the glmnet package. The optimal regularization parameter (lambda.1se) was determined through 10-fold cross-validation to ensure model parsimony. Figure 1A displays the cross-validation curve showing binomial deviance vs. log(lambda). The LASSO procedure reduced 40 initial variables to 14 predictors with non-zero coefficients (Figure 1B): hypertension, COPD, ABSI, smoking status, NFS, malignancy, age, diabetes, prediabetes, sex, race, total bilirubin, family PIR, and albumin.

### Performance of Machine Learning Models

Fifteen ML algorithms were evaluated on both training and validation sets. Supplementary Figure 3 presents forest plots comparing AUC values with 95% CIs. In the training set (Supplementary Figure 3A), LightGBM and CatBoost achieved perfect AUC of 1.0 (95% CI: 1.0-1.0), suggesting potential overfitting. Random Forest (AUC = 0.854, 95% CI: 0.836-0.872) and SVM with radial kernel (AUC = 0.842, 95% CI: 0.824-0.860) showed more conservative but generalizable performance. XGBoost achieved AUC of 0.797, while linear models showed lower performance (logistic Regression: 0.601, LASSO: 0.593). In the validation set (Supplementary Figure 3B), Random Forest emerged as the top performer with AUC of 0.860 (95% CI: 0.833-0.887), demonstrating excellent generalization. The SVM (AUC = 0.838), CatBoost (AUC = 0.829), and LightGBM (AUC = 0.827) followed closely. The consistency between training and validation performance indicated minimal overfitting.

Supplementary Figure 4 illustrates comprehensive performance metrics. Random Forest exhibited balanced performance with 92.7% accuracy, 100% specificity, 100% positive predictive value, 92.1% negative predictive value, F1 score of 0.649, and Youden’s index of 0.481.

**Figure 2.**
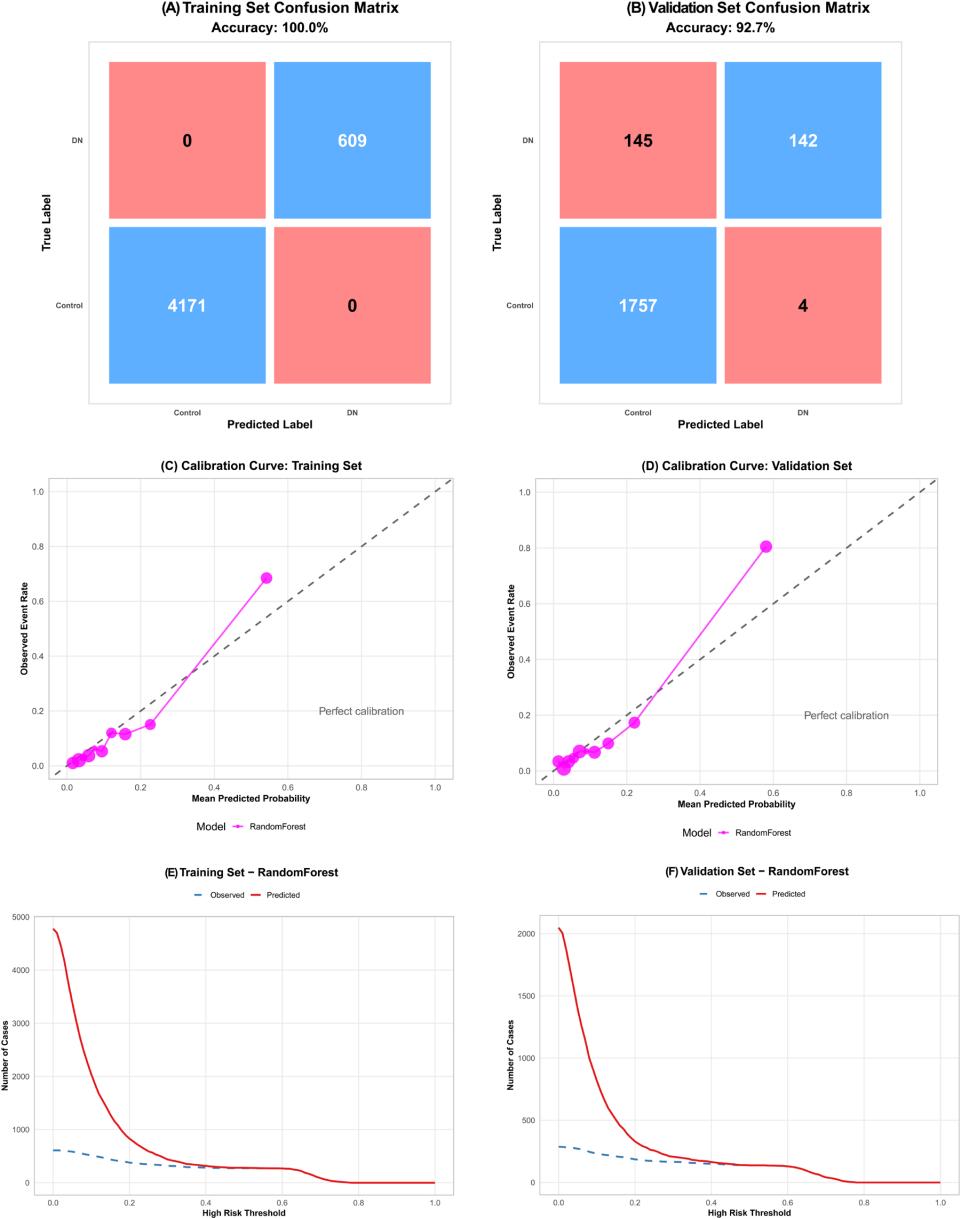
Detailed assessment of Random Forest model performance for CVD prediction. (A and B) Confusion matrices showing classification results in training (A) and validation (B) sets, with the model achieving 100% accuracy in training and 92.7% in validation. (C-D) Calibration curves comparing predicted probabilities (x-axis) with observed event rates (y-axis) for training (C) and validation (D) sets, with the diagonal dashed line representing perfect calibration. (E-F) Clinical impact curves showing the number of predicted (red solid line) vs. observed (blue dashed line) CVD cases across different risk thresholds for training (E) and validation (F) sets, demonstrating good clinical utility.

Figure 2 presents detailed Random Forest assessment. Confusion matrices (Figure 2A and B) showed excellent classification: training set achieved 100% accuracy (4171 controls, 609 CVD cases correctly identified); validation set maintained 92.7% accuracy with 99.8% specificity and 49.5% sensitivity. Calibration curves (Figure 2C and D) closely followed the diagonal reference line in both sets, indicating excellent calibration across predicted probabilities. Clinical impact curves (Figure 2E and F) demonstrated close alignment between observed and predicted case numbers across risk thresholds, confirming good clinical utility.

### Relative Significance of Factors in Machine Learning

**Figure 3.**
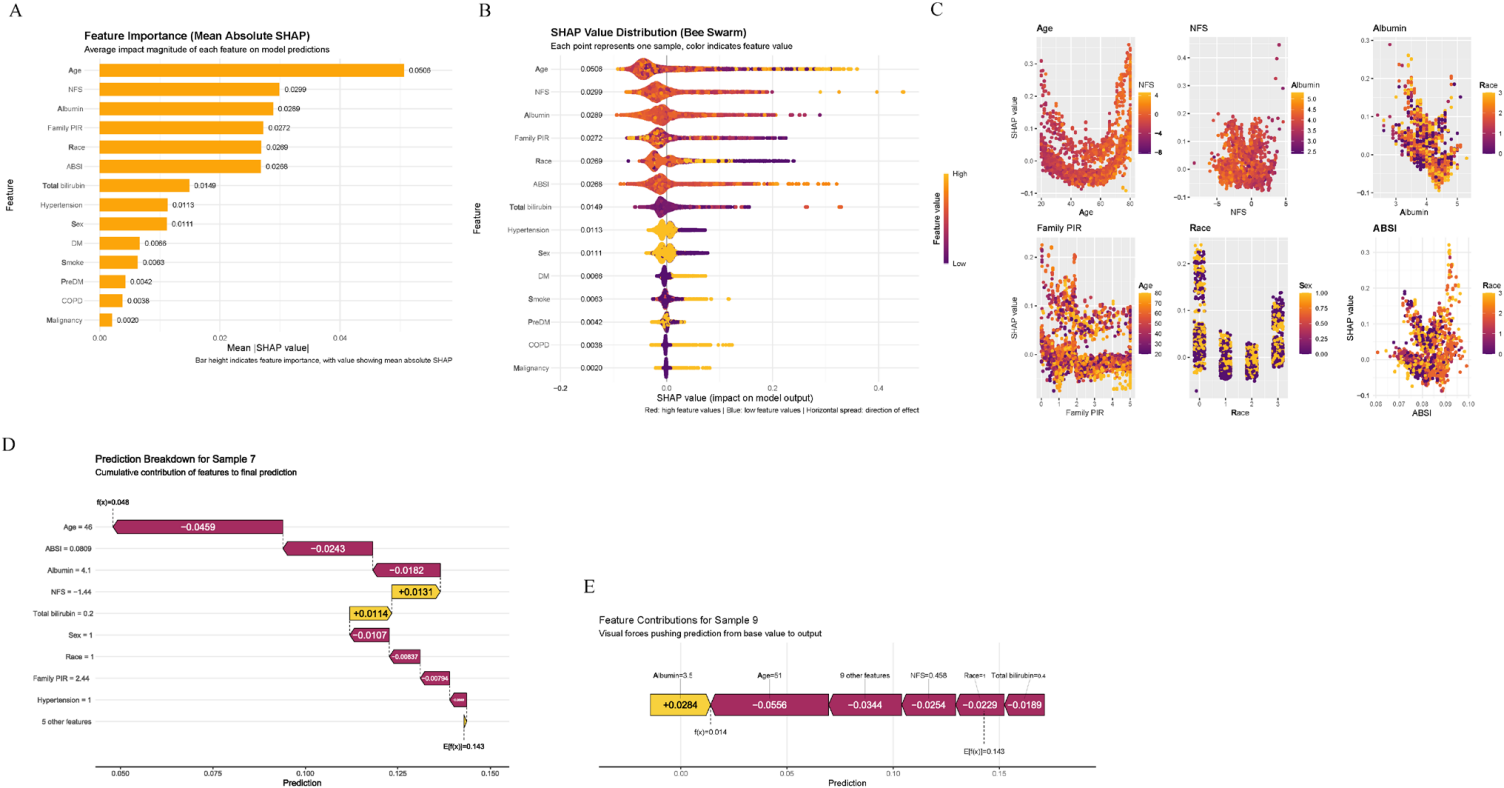
SHapley Additive exPlanations (SHAP) analysis revealing feature contributions in the Random Forest model. (A) Feature importance bar plot showing mean absolute SHAP values, with NFS emerging as the most influential predictor. (B) Bee swarm plot displaying SHAP value distributions, where each point represents 1 sample colored by feature value (red = high, blue = low), revealing feature-outcome relationships. (C) Dependence plots for the top 6 predictors illustrating non-linear relationships between feature values and SHAP contributions. (D) Waterfall plot showing cumulative feature contributions for a representative sample, demonstrating how individual features push the prediction from baseline (E [f(x)] = 0.143) to final output (f(x) = 0.014). (E) Force plot providing an alternative visualization of feature contributions for the same sample, with features pushing the prediction toward higher (red) or lower (blue) CVD risk.

To enhance interpretability, SHAP analysis was employed to quantify feature contributions to predictions. Figure 3 presents comprehensive SHAP-based feature importance analysis. The SHAP feature importance bar plot (Figure 3A) revealed mean absolute SHAP values, with age emerging as the most influential variable (0.0506), followed by NFS (0.0299), albumin (0.0289), family PIR (0.0272), and race (0.0269). The ABSI contributed moderately (0.0268), while traditional cardiovascular risk factors including hypertension, diabetes, and smoking showed lower contributions. The SHAP bee swarm plot (Figure 3B) provided deeper insights into feature-outcome relationships. Increasing NFS values were consistently associated with higher predicted CVD probability, while increasing albumin levels were associated with lower CVD risk. Age showed clear positive correlation with CVD risk. Family PIR demonstrated complex non-linear relationships. Feature dependence plots (Figure 3C) illustrated detailed non-linear relationships for top predictors. The NFS demonstrated threshold effects with increasing SHAP values above −1.0. Albumin showed protective effects plateauing below 3.5 g/dL. Age exhibited nearly linear positive association after 50 years. Individual prediction explanations were visualized using waterfall plots (Figure 3D) and force plots (Figure 3E), which demonstrated how combinations of clinical features influenced patient-specific CVD risk predictions.

These SHAP analyses revealed that age, liver-specific markers (NFS and albumin), and metabolic factors dominated CVD risk prediction in MAFLD patients, underscoring the importance of comprehensive metabolic and liver function assessment in cardiovascular risk stratification.

## Discussion

This study successfully developed and validated ML models to predict CVD risk in patients with MAFLD using NHANES 2017-2023 data. The Random Forest model emerged as the optimal predictor, achieving an AUC of 0.854 (95% CI: 0.836-0.872) in training and 0.860 (95% CI: 0.833-0.887) in validation sets. Most notably, liver-specific markers—particularly the NFS and albumin levels—demonstrated superior predictive capacity compared to traditional cardiovascular risk factors, challenging conventional risk assessment paradigms. The model exhibited excellent calibration across the full spectrum of predicted probabilities and demonstrated substantial clinical utility across various risk thresholds, suggesting its potential for immediate clinical implementation.

The Random Forest model (AUC: 0.860) demonstrates superior predictive performance compared to previously established models. Abeles et al^19^ developed a NAFLD-specific cardiovascular risk score achieving an AUC of 0.84 in derivation and 0.77 in validation cohorts, while the approach maintains consistently high performance suggesting better generalizability. Ichikawa et al^20^ showed that adding coronary artery calcium scores to clinical risk factors increased the C-statistic from 0.677 to 0.739, still substantially lower than the model’s performance. Goldman et al^21^ achieved an AUC above 0.82 for NAFLD and fibrosis prediction using ML, though focusing on liver rather than cardiovascular outcomes. Traditional cardiovascular risk scores demonstrate significant limitations in NAFLD populations. Gheorghe et al^22^ noted conventional scores underestimate cardiovascular risk in NAFLD patients, while Kasapoglu et al^23^ found Framingham scoring failed to capture the full risk profile, particularly regarding liver-specific biomarkers. The model’s enhanced discriminative ability stems from integrating MAFLD-specific predictors, with NFS emerging as the most influential predictor, consistent with Rivera-Esteban et al’s^24^ findings on liver stiffness predicting outcomes. The ML approach effectively captures complex non-linear relationships, addressing the multisystemic nature of MAFLD described by Targher et al,^25^ involving hepatic insulin resistance, atherogenic dyslipidemia, and systemic inflammation. The novelty of applying this framework to NHANES data for MAFLD-CVD prediction represents a significant methodological advancement.

The NFS’s emergence as the strongest predictor highlights hepatic fibrosis’s fundamental role in cardiovascular pathogenesis.^26^^,^^27^ The NFS incorporates age, BMI, diabetes, AST/ALT ratio, platelets, and albumin, capturing both metabolic dysfunction and hepatic structural changes. The SHAP analysis revealed a threshold effect at NFS values above −1.0, identifying a clear intervention point for aggressive risk modification. This aligns with evidence linking fibrosis progression to subclinical atherosclerosis markers including increased carotid intima-media thickness and coronary calcium scores.^28^^,^^29^ Albumin’s protective effect, plateauing below 3.5 g/dL, reflects its role as a marker of liver synthetic function, inflammation, and nutrition.^30^^-^^32^ Beyond hepatic synthesis, albumin possesses direct cardioprotective properties including antioxidant and anti-inflammatory effects. Hypoalbuminemia in MAFLD may indicate advanced disease, malnutrition, or chronic inflammation—all independently increasing cardiovascular risk. Age demonstrated a nearly linear positive association after 50 years, representing cumulative metabolic insults and age-related cardiovascular changes. The ABSI’s contribution over BMI highlights the importance of visceral adiposity in MAFLD-CVD risk,^33^^,^^34^ with values above 0.08 associated with increased risk. The family PIR’s inclusion reveals socioeconomic dimensions often overlooked, while racial disparities reflect complex interactions between genetic susceptibility, environmental exposures, and healthcare access.

Random Forest’s superior performance stems from its ability to capture complex non-linear relationships and high-order interactions without explicit specification.^35^^,^^36^ The algorithm’s bootstrap aggregation provides inherent regularization, preventing overfitting while maintaining accuracy—demonstrated by consistent performance across datasets. Its robustness to outliers and mixed data types makes it particularly suited for heterogeneous clinical data. The overfitting in LightGBM/CatBoost (perfect training AUC declining to 0.827-0.829 in validation) highlights gradient boosting’s memorization risk with imbalanced data. Random Forest’s parallel tree construction provides natural regularization through averaging. The model’s moderate sensitivity (49.5%) vs. high specificity (99.8%) reflects important clinical considerations. While missing half of CVD cases raises concerns, the high negative predictive value (92.1%) and reality that universal intensive prevention isn’t feasible make this acceptable. High specificity ensures identified high-risk patients truly warrant aggressive intervention. False negatives lead to risk of delayed intervention but all MAFLD patients receive basic preventive care per guidelines. False positives lead to intensive monitoring potentially benefiting these elevated-risk patients. Excellent calibration suggests probability estimates can guide risk-stratified intervention intensity. While logistic regression’s interpretability has merit, the 26% AUC improvement with Random Forest justifies additional complexity for high-stakes assessment, especially as modern electronic health records (EHRs) increasingly support complex algorithm deployment.

The NHANES dataset (2017-2023) includes 6828 MAFLD adults, ensuring broad generalizability through rigorous sampling and standardized protocols. Systematically comparing 15 algorithms ensures identification of the truly optimal approach rather than accepting predetermined methods. SHAP analysis addresses the “black box” concern limiting ML adoption. By quantifying feature contributions, SHAP transforms Random Forest into an interpretable clinical tool. Comprehensive visualizations provide clear, practical insights for each patient, which helps clinicians accept them and supports shared decision-making. Robust validation beyond discrimination metrics included calibration assessment and decision curve analysis, directly evaluating clinical utility. Appropriate handling of class imbalance and missing data through Random Forest imputation preserves variable relationships while maintaining real-world applicability.

The cross-sectional design precludes causal inference and temporal relationship assessment. While strong associations between liver markers and CVD were identified, causation vs. confounding cannot be determined. The inability to capture disease trajectories limits prediction of incident vs. prevalent disease. Although a rigorous methodology with internal cross-validation for hyperparameter optimization was employed and a completely independent validation set was maintained for final evaluation, using a 3-way data split (training/validation/test sets) would represent a more conservative approach for complex model development. Future studies with larger sample sizes may benefit from implementing such 3-way splits to further minimize potential optimism in performance estimates. Nevertheless, the consistency between the training and validation performance, along with excellent calibration in both cohorts, suggests minimal overfitting in the final models. Reliance on clinical scores rather than imaging-based assessment is significant given evolving standards. The NFS may misclassify fibrosis severity compared to elastography or biopsy. Lack of longitudinal validation prevents assessment of true predictive performance for incident events. Self-reported outcomes may introduce misclassification bias. Limited biomarker availability excludes established markers like high-sensitivity CRP, troponin, and NT-proBNP. Absence of genetic data precludes polygenic risk score incorporation. Medication data limitations prevent full adjustment for treatment effects.

In conclusion, this study successfully developed and validated a ML model for CVD prediction in MAFLD patients, achieving superior performance compared to traditional risk scores. The Random Forest algorithm demonstrated excellent calibration and clinical utility, enabling implementation through existing EHRs using readily available clinical variables. Most significantly, liver-specific markers—particularly NFS and albumin-dominated cardiovascular risk prediction, surpassing traditional risk factors. This paradigm-shifting finding challenges conventional separate assessment of hepatic and cardiovascular risk, supporting an integrated liver-heart axis framework. The prominence of fibrosis underscores the need for routine hepatic assessment in cardiovascular risk stratification.

## Supplementary Materials

Supplementary Material

## Figures and Tables

**Table 1.** Baseline Characteristics of Study Participants with MAFLD Stratified by Training and Validation Sets

**Variables**	**Training (n = 4780)**	**Validation (n = 2048)**	***P***
Age, mean ± SD	53.19 ± 16.19	53.58 ± 15.76	.361
BMI, mean ± SD	32.91 ± 7.13	32.86 ± 7.30	.809
Waist circumference, mean ± SD	108.82 ± 15.06	108.85 ± 15.26	.937
HbA1c, mean ± SD	6.08 ± 1.23	6.13 ± 1.36	.133
Alkaline phosphatase, mean ± SD	81.00 ± 24.75	81.62 ± 26.38	.368
Albumin, mean ± SD	4.03 ± 0.33	4.04 ± 0.33	.363
Uric acid, mean ± SD	5.74 ± 1.47	5.74 ± 1.49	.891
HDL cholesterol, mean ± SD	49.27 ± 14.14	49.59 ± 14.80	.393
SBP, mean ± SD	126.45 ± 18.31	127.12 ± 17.86	.162
DBP, mean ± SD	76.29 ± 11.36	76.66 ± 10.94	.222
eGFR, mean ± SD	91.90 ± 23.01	91.44 ± 22.68	.447
ABSI, mean ± SD	0.08 ± 0.00	0.08 ± 0.00	.836
eGDR, mean ± SD	6.14 ± 2.53	6.10 ± 2.56	.502
Family PIR, M (Q_1_, Q_3_)	2.28 (1.36, 3.79)	2.33 (1.37, 3.85)	.525
ALT, M (Q_1_, Q_3_)	20.00 (14.00, 29.00)	20.00 (15.00, 30.00)	.104
AST, M (Q_1_, Q_3_)	20.00 (16.00, 25.00)	20.00 (16.00, 25.00)	.189
Total bilirubin, M (Q_1_, Q_3_)	0.40 (0.30, 0.50)	0.40 (0.30, 0.60)	.147
Gamma glutamyl transferase, M (Q_1_, Q_3_)	24.00 (17.00, 38.00)	25.00 (18.00, 38.00)	.058
Creatinine, M (Q_1_, Q_3_)	0.85 (0.71, 1.01)	0.85 (0.72, 1.01)	.329
Blood urea nitrogen, M (Q_1_, Q_3_)	14.00 (11.00, 18.00)	14.00 (12.00, 18.00)	.082
Total cholesterol, M (Q_1_, Q_3_)	186.00 (161.00, 215.00)	188.00 (161.00, 215.00)	.350
Hs C-reactive protein, M (Q_1_, Q_3_)	2.69 (1.23, 5.55)	2.60 (1.20, 5.19)	.142
WBC, M (Q_1_, Q_3_)	7.20 (6.00, 8.80)	7.20 (6.07, 8.60)	.614
Lymphocyte, M (Q_1_, Q_3_)	2.20 (1.80, 2.70)	2.20 (1.70, 2.70)	.906
Monocyte, M (Q_1_, Q_3_)	0.60 (0.50, 0.70)	0.60 (0.50, 0.70)	.758
Neutrophils, M (Q_1_, Q_3_)	4.20 (3.20, 5.30)	4.10 (3.20, 5.20)	.480
Platelet, M (Q_1_, Q_3_)	242.00 (206.00, 285.00)	238.00 (198.00, 284.00)	.019
FIB4, M (Q_1_, Q_3_)	0.97 (0.63, 1.39)	0.99 (0.66, 1.43)	.086
NFS, M (Q_1_, Q_3_)	−1.07 (−2.18, 0.01)	−0.98 (−2.07, 0.06)	.047
Race, n (%)			.809
Mexican American	730 (15.27)	331 (16.16)	
Non-Hispanic Black	1025 (21.44)	439 (21.44)	
Non-Hispanic White	1688 (35.31)	708 (34.57)	
Other	1337 (27.97)	570 (27.83)	
Sex, n (%)			.086
Female	2295 (48.01)	937 (45.75)	
Male	2485 (51.99)	1111 (54.25)	
Education, n (%)			.875
Less than high school	950 (19.87)	418 (20.41)	
High school graduate	1158 (24.23)	495 (24.17)	
Above high school	2672 (55.90)	1135 (55.42)	
Malignancy, n (%)			.811
No	4292 (89.79)	1835 (89.60)	
Yes	488 (10.21)	213 (10.40)	
Smoke, n (%)			.872
Never	2706 (56.61)	1156 (56.45)	
Former	1298 (27.15)	567 (27.69)	
Current	776 (16.23)	325 (15.87)	
Diabetes, n (%)			.428
No	3367 (70.44)	1423 (69.48)	
Yes	1413 (29.56)	625 (30.52)	
Prediabetes, n (%)			.926
No	2746 (57.45)	1179 (57.57)	
Yes	2034 (42.55)	869 (42.43)	
CKD, n (%)			.665
No	3780 (79.08)	1610 (78.61)	
Yes	1000 (20.92)	438 (21.39)	
COPD, n (%)			.757
No	4344 (90.88)	1866 (91.11)	
Yes	436 (9.12)	182 (8.89)	
Hypertension, n (%)			.737
0	2159 (45.17)	916 (44.73)	
1	2621 (54.83)	1132 (55.27)	
Alcohol user, n (%)			.460
Never	441 (9.23)	165 (8.06)	
Former	1066 (22.30)	442 (21.58)	
Mild	1708 (35.73)	757 (36.96)	
Moderate	703 (14.71)	299 (14.60)	
Heavy	862 (18.03)	385 (18.80)	

SD, standard deviation; BMI, body mass index; HbA1c, glycated hemoglobin; HDL, high-density lipoprotein; SBP, systolic blood pressure; DBP, diastolic blood pressure; eGFR, estimated glomerular filtration rate; ABSI, a body shape index; eGDR, estimated glucose disposal rate; PIR, income to poverty ratio; ALT, alanine aminotransferase; AST, aspartate aminotransferase; Q1, quartile 25%; Q3, quartile 75%; WBC, white blood count; FIB4, fibrosis index based on four factors; NFS, non-alcoholic fatty liver disease fibrosis score; CKD, chronic kidney disease; COPD, chronic obstructive pulmonary disease.

## Data Availability

The data that support the findings of this study are available on request from the corresponding author.

## References

[1] Eslam M Sanyal AJ George J International Consensus Panel. MAFLD: A consensus-driven proposed nomenclature for metabolic associated fatty liver disease. Gastroenterology. 2020;158(7):1999 2014.e1. (doi: 10.10.1053/j.gastro.2019.11.312) 32044314

[2] Eslam M Newsome PN Sarin SK A new definition for metabolic dysfunction-associated fatty liver disease: an international expert consensus statement. J Hepatol. 2020;73(1):202 209. (doi: 10.10.1016/j.jhep.2020.03.039) 32278004

[3] Nguyen VH Le MH Cheung RC Nguyen MH. Differential Clinical Characteristics and Mortality Outcomes in Persons with NAFLD and/or MAFLD. Clin Gastroenterol Hepatol : Off Clin Pract J Am Gastroenterological Assoc. 2021;19(10):2172 2181.e6. (doi: 10.10.1016/j.cgh.2021.05.029) 34033923

[4] Sarin SK Kumar M Eslam M Liver diseases in the Asia-Pacific region: a Lancet Gastroenterology & Hepatology Commission. Lancet Gastroenterol Hepatol. 2020;5(2):167 228. (doi: 10.10.1016/S2468-1253(19)30342-5) 31852635 PMC7164809

[5] Eslam M Sarin SK Wong VW The Asian Pacific Association for the Study of the Liver clinical practice guidelines for the diagnosis and management of metabolic associated fatty liver disease. Hepatol Int. 2020;14(6):889 919. (doi: 10.10.1007/s12072-020-10094-2) 33006093

[6] Chan KE Ng CH Fu CE The spectrum and impact of metabolic dysfunction in MAFLD: A longitudinal cohort analysis of 32,683 overweight and obese individuals. Clin Gastroenterol Hepatol : Off Clin Pract J Am Gastroenterological Assoc. 2023;21(10):2560 2569.e15. (doi: 10.10.1016/j.cgh.2022.09.028) 36202348

[7] Şahintürk Y Köker G Koca N Metabolic dysfunction-associated fatty liver disease and fibrosis status in patients with type 2 diabetes treated at internal medicine clinics: Türkiye DAHUDER awareness of fatty liver disease (TR-DAFLD) study. Turk J Gastroenterol. 2024;35(8):643 650. (doi: 10.10.5152/tjg.2024.24045) 39150440 PMC11363181

[8] Chen B Wang H Xu S Association between the triglyceride glucose-body mass index and mortality risk in cardiovascular disease populations: a longitudinal cohort study. BMC Public Health. 2025;25(1):822. (doi: 10.10.1186/s12889-025-22018-6) PMC11871752 40022003

[9] Chen X Chen Z Jiang L Huang J Zhu Y Lin S. MAFLD is associated with increased all-cause mortality in low cardiovascular-risk individuals but not in intermediate to high-risk individuals. Nutr Metab Cardiovasc Dis. 2023;33(2):376 384. (doi: 10.10.1016/j.numecd.2022.11.007) 36599780

[10] Zhang G Dong S Wang L. Construction of a machine learning-based risk prediction model for depression in middle-aged and elderly patients with cardiovascular metabolic diseases in China: a longitudinal study. BMC Public Health. 2025;25(1):1904. (doi: 10.10.1186/s12889-025-23075-7) 40410764 PMC12101033

[11] Obermeyer Z Emanuel EJ. Predicting the future - big data, machine learning, and clinical medicine. N Engl J Med. 2016;375(13):1216 1219. (doi: 10.10.1056/NEJMp1606181) 27682033 PMC5070532

[12] Zou H Zhao F Lv X Ma X Xie Y. Development and validation of a new nomogram to screen for MAFLD. Lipids Health Dis. 2022;21(1):133. (doi: 10.10.1186/s12944-022-01748-1) PMC9730620 36482400

[13] Karlas T Petroff D Sasso M Individual patient data meta-analysis of controlled attenuation parameter (CAP) technology for assessing steatosis. J Hepatol. 2017;66(5):1022 1030. (doi: 10.10.1016/j.jhep.2016.12.022) 28039099

[14] Kurexi A Peng J Yao J Wang L Wang Q. Association of “a body shape index” with the risk of developing colorectal cancer in U.S. patients with metabolic syndrome: evidence from the NHANES 1999-2018. BMC Gastroenterol. 2024;24(1):447. (doi: 10.10.1186/s12876-024-03537-9) PMC11613469 39627686

[15] Inker LA Eneanya ND Coresh J New creatinine- and cystatin C-based equations to estimate GFR without race. N Engl J Med. 2021;385(19):1737 1749. (doi: 10.10.1056/NEJMoa2102953) 34554658 PMC8822996

[16] Dong B Chen Y Yang X Estimated glucose disposal rate outperforms other insulin resistance surrogates in predicting incident cardiovascular diseases in cardiovascular-kidney-metabolic syndrome stages 0-3 and the development of a machine learning prediction model: a nationwide prospective cohort study. Cardiovasc Diabetol. 2025;24(1):163. (doi: 10.10.1186/s12933-025-02729-1) PMC12004813 40241176

[17] Treeprasertsuk S Björnsson E Enders F Suwanwalaikorn S Lindor KD. NAFLD fibrosis score: a prognostic predictor for mortality and liver complications among NAFLD patients. World J Gastroenterol. 2013;19(8):1219 1229. (doi: 10.10.3748/wjg.v19.i8.1219) 23482703 PMC3587478

[18] Tang F Ishwaran H. Random forest missing data algorithms. Stat Anal Data Min. 2017;10(6):363 377. (doi: 10.10.1002/sam.11348) 29403567 PMC5796790

[19] Abeles RD Mullish BH Forlano R Derivation and validation of a cardiovascular risk score for prediction of major acute cardiovascular events in non-alcoholic fatty liver disease; the importance of an elevated mean platelet volume. Aliment Pharmacol Ther. 2019;49(8):1077 1085. (doi: 10.10.1111/apt.15192) 30836450 PMC6519040

[20] Ichikawa K Hansen S Manubolu VS Prognostic value of coronary artery calcium score for the prediction of atherosclerotic cardiovascular disease in participants with suspected nonalcoholic hepatic steatosis: results from the multi-ethnic study of atherosclerosis. Am Heart J. 2023;265:104 113. (doi: 10.10.1016/j.ahj.2023.07.008) 37517431 PMC10592252

[21] Goldman O Ben-Assuli O Rogowski O Non-alcoholic fatty liver and liver fibrosis predictive analytics: risk prediction and machine learning techniques for improved preventive medicine. J Med Syst. 2021;45(2):22. (doi: 10.10.1007/s10916-020-01693-5) 33426569

[22] Gheorghe L Nemteanu R Clim A Botnariu GE Costache II Plesa A. Risk scores for prediction of major cardiovascular events in non-alcoholic fatty liver disease: A no man's land? Life (Basel, Switzerland). 2023;13(4):857. (doi: 10.10.3390/life13040857) 37109386 PMC10146692

[23] Kasapoglu B Turkay C Yalcın KS Carlioglu A Koktener A. Role of γ-glutamyl transferase levels in prediction of high cardiovascular risk among patients with non-alcoholic fatty liver disease. Indian J Med Res. 2016;143(1):30 36. (doi: 10.10.4103/0971-5916.178585) 26997011 PMC4822365

[24] Rivera-Esteban J Pons M Planas A Prediction of clinical events by liver stiffness and chronic kidney disease by NAFLD in patients with type-2 diabetes. Gastroenterol Hepatol. 2023;46(9):682 691. (doi: 10.10.1016/j.gastrohep.2022.11.001) 36435379

[25] Targher G Corey KE Byrne CD. NAFLD, and cardiovascular and cardiac diseases: factors influencing risk, prediction and treatment. Diabetes Metab. 2021;47(2):101215. (doi: 10.10.1016/j.diabet.2020.101215) 33296704

[26] Liu X Zhang HJ Fang CC Association between noninvasive liver fibrosis scores and heart failure in a general population. J Am Heart Assoc. 2024;13(22):e035371. (doi: 10.10.1161/JAHA.123.035371) PMC11681418 39508146

[27] Zhang J Li L Lin L Prognostic value of FIB-4 and NFS for cardiovascular events in patients with and without NAFLD. BMC Public Health. 2025;25(1):2747. (doi: 10.10.1186/s12889-025-23883-x) PMC12341116 40797198

[28] Zhang H Lin F Miao M Yu C Guo W. Liver fibrosis increases the risk of subclinical atherosclerosis in patients with MASLD: a cross-sectional and longitudinal study. Nutr Metab (Lond). 2025;22(1):68. (doi: 10.10.1186/s12986-025-00951-y) PMC12218835 40598295

[29] Arai T Atsukawa M Tsubota A Liver fibrosis is associated with carotid atherosclerosis in patients with liver biopsy-proven nonalcoholic fatty liver disease. Sci Rep. 2021;11(1):15938. (doi: 10.10.1038/s41598-021-95581-8) PMC8342487 34354193

[30] Duran-Güell M Garrabou G Flores-Costa R Essential role for albumin in preserving liver cells from TNFα-induced mitochondrial injury. FASEB J. 2023;37(3):e22817. (doi: 10.10.1096/fj.202201526R) 36809676

[31] Gremese E Bruno D Varriano V Perniola S Petricca L Ferraccioli G. Serum albumin levels: A biomarker to be repurposed in different disease settings in clinical practice. J Clin Med. 2023;12(18):6017. (doi: 10.10.3390/jcm12186017) 37762957 PMC10532125

[32] Carvalho JR Verdelho Machado M. New insights about albumin and liver disease. Ann Hepatol. 2018;17(4):547 560. (doi: 10.10.5604/01.3001.0012.0916) 29893696

[33] Lee H Chung HS Kim YJ Association between body shape index and risk of mortality in the United States. Sci Rep. 2022;12(1):11254. (doi: 10.10.1038/s41598-022-15015-x) PMC9253149 35788633

[34] Kuang M Sheng G Hu C Lu S Peng N Zou Y. The value of combining the simple anthropometric obesity parameters, body mass index (BMI) and a Body Shape Index (ABSI), to assess the risk of non-alcoholic fatty liver disease. Lipids Health Dis. 2022;21(1):104. (doi: 10.10.1186/s12944-022-01717-8) PMC9585710 36266655

[35] Goldstein BA Polley EC Briggs FB. Random forests for genetic association studies. Stat Appl Genet Mol Biol. 2011;10(1):32. (doi: 10.10.2202/1544-6115.1691) PMC3154091 22889876

[36] Chen R-C Dewi C Huang S-W Caraka RE. Selecting critical features for data classification based on machine learning methods. J Big Data. 2020;7(1):52. (doi: 10.10.1186/s40537-020-00327-4)

