# Supplementary material for: Development and Validation of a Machine Learning–Based Prediction Model for Cardiovascular Disease in Patients with Metabolic Dysfunction–Associated Fatty Liver Disease
Source: Turk J Gastroenterol. 2026 Feb 13;37(4):471–82. doi: 10.5152/tjg.2026.25611 (PMC13047333; doi:10.5152/tjg.2026.25611)
Supplement: Supplementary Material [file supplementary_material.pdf]

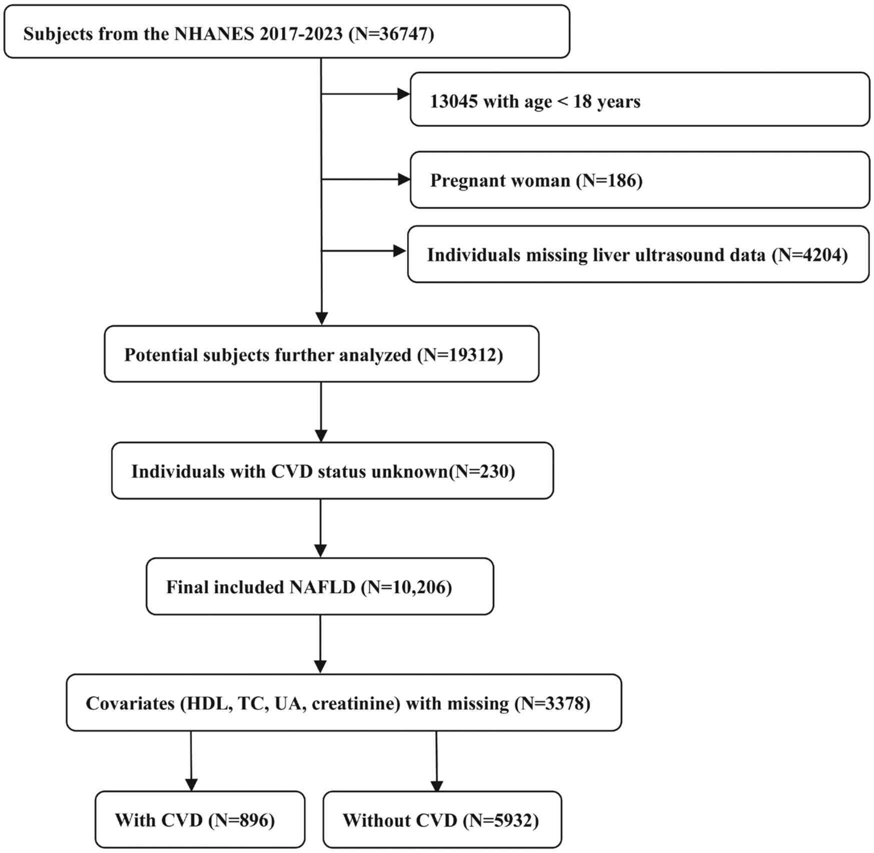

Supplementary Figure 1. Flow diagram of participant selection from NHANES 2017-2023. The study screened participants from the National Health and Nutrition Examination Survey (NHANES) spanning three cycles (2017-2018, 2019-2020, and 2021-2023). Among 6,828 adults, 896 (13.1%) had prevalent CVD and 5,932 (86.9%) did not have CVD.

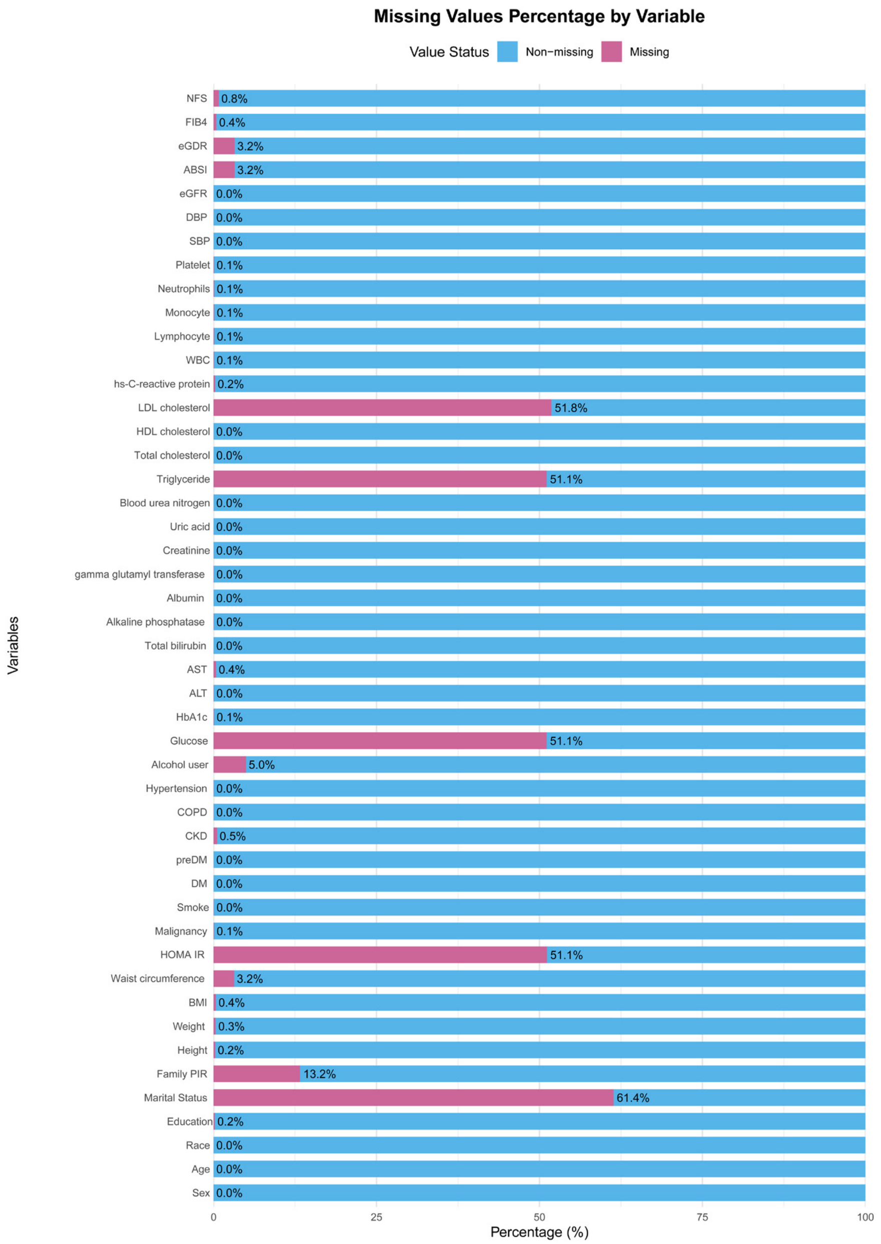

Supplementary Figure 2. Distribution of missing values across all variables in the dataset. The horizontal bar chart displays the percentage of missing values for each variable, with non-missing data shown in blue and missing data in red. Most variables demonstrated excellent completeness with less than 1% missing values. Notable exceptions included marital status (61.4%), HOMA-IR (51.1%), fasting glucose (51.1%), fasting lipid panels (51.1-51.8%), and family poverty-income ratio (13.2%). Variables with substantial missingness were evaluated for clinical importance before imputation using the random forest method.

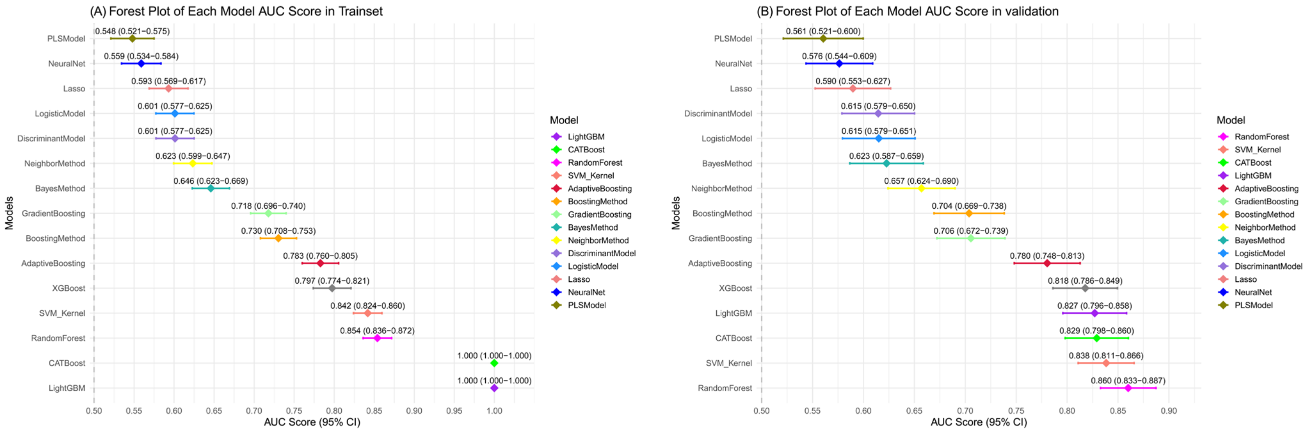

Supplementary Figure 3. Forest plots comparing area under the receiver operating characteristic curve (AUC) values across fifteen machine learning models. (A) Training set performance showing AUC values with 95% confidence intervals. (B) Validation set performance revealing Random Forest as the top performer.

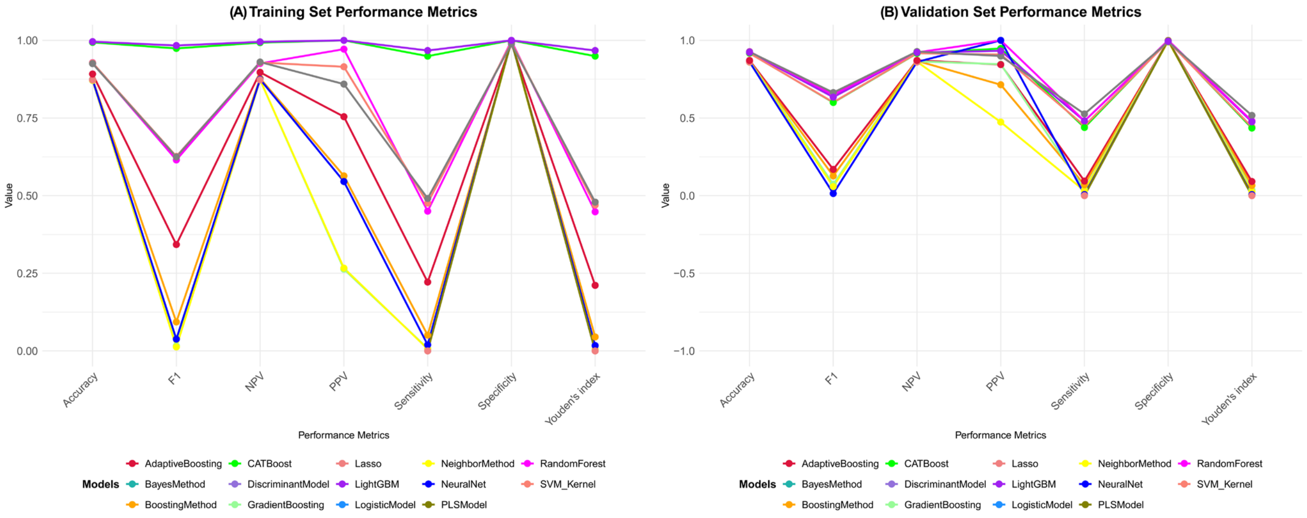

Supplementary Figure 4. Comprehensive performance metrics for all fifteen machine learning models. Radar plots displaying seven performance metrics (Accuracy, F1 score, NPV, PPV, Sensitivity, Specificity, and Youden's index) for (A) training set and (B) validation set.
